# Unveiling the Uncommon: Roseomonas gilardii Bacteremia in a 10-Month Infant Presenting With Febrile Seizure

**DOI:** 10.7759/cureus.87503

**Published:** 2025-07-08

**Authors:** Prashanth Purushotham, Ashoka Mahapatra, Nida Jafri, Amit Satapathy

**Affiliations:** 1 Microbiology, All India Institute of Medical Sciences, Bhubaneswar, Bhubaneswar, IND; 2 Pediatrics, All India Institute of Medical Sciences, Bhubaneswar, Bhubaneswar, IND

**Keywords:** bacteraemia, fever, neonate, roseomonas gilardii, seizure

## Abstract

*Roseomonas gilardii* (*R. gilardii*), a Gram-negative coccobacillus, is an opportunistic pathogen most commonly isolated from immunocompromised children, especially those with underlying malignancies. In this case report, we present a non-immunocompromised 10-month-old infant diagnosed with *R. gilardii* bacteremia. Febrile seizures are a common neurological presentation in young children and are often associated with underlying infections such as bacteremia. While pathogens like *Staphylococcus aureus* and *Salmonella* species are commonly implicated, a proportion of cases remain without an identified causative organism. Although *R. gilardii* is typically associated with immunocompromised states, in this case, it was isolated from a previously healthy child. The primary clinical features included recurrent seizures, thrombocytopenia, and mild hearing loss. The isolation of *R. gilardii* in an immunocompetent infant is exceptionally rare, making this report clinically significant. The organism was identified using the VITEK® 2 Compact system (bioMérieux, Marcy l’Étoile, France), which enables rapid and accurate identification of rare, fastidious, or atypical organisms like *R.gilardii* by utilizing an extensive biochemical database and automated card-based technology, further enhancing diagnostic precision where conventional methods may be inconclusive. Antibiotic susceptibility testing showed sensitivity to ceftazidime, ceftriaxone, cefoperazone-sulbactam, cefepime, aztreonam, imipenem, meropenem, amikacin, gentamicin, ciprofloxacin, and levofloxacin. The patient was successfully treated with ceftriaxone. Although the mortality rate associated with *Roseomonas* species infections is low, this report highlights the importance of microbiologicalawareness, accurate identification, and appropriate antimicrobial therapy in pediatric cases involving rare pathogens. This is particularly important in pediatric and immunocompetent patients, where timely and accurate identification of unusual pathogens can significantly influence clinical decisions and outcomes.

## Introduction

Febrile seizures, defined as seizures triggered by fever, commonly occur in children between six months and five years of age, with a peak incidence between 12 and 18 months [1]. Bacteremia is frequently detected when blood cultures are routinely performed in children admitted with febrile seizures [2]. Bacteremia accounts for approximately 21.8% of febrile seizure cases, with *Staphylococcus aureus* and *Salmonella* species being the most commonly identified organisms. However, approximately 12.5% of cases remain without an identified pathogen [3]. Therefore, accurate bacterial identification is essential for guiding appropriate therapy.

*Roseomonas gilardii* (*R. gilardii*) is a pink-pigmented, Gram-negative coccobacillus of the genus *Roseomonas*, first described in 1993 [4]. Among its species, *R. gilardii* and *R. mucosa* are the only two known to be pathogenic in humans. *R. gilardii* is an opportunistic pathogen frequently isolated from immunocompromised pediatric patients, particularly those with malignancies [5]. Here, we report a rare case of febrile seizures due to *R. gilardii* in a non-immunocompromised child. To our knowledge, this is one of only five reported pediatric cases of *R. gilardii* infection, and the first reported case from India [5].

## Case presentation

A 10-month-old male infant was brought to the pediatric outpatient department with a three-day history of fever with chills and rigors, along with a single episode of generalized abnormal movements involving all four limbs lasting two minutes, associated with spontaneous urination and followed by drowsiness for 30-60 minutes. A similar episode had occurred the previous month and was treated with levetiracetam at 27 mg/kg at a local hospital. Birth history was unremarkable except for neonatal jaundice on day 3, treated with phototherapy. Immunizations were up to date.

On examination, the heart rate was 128 bpm, respiratory rate 36/min, temperature 101.3°F, and SpO₂ 98%. The child appeared ill and irritable, but a neurological exam revealed no signs of meningeal irritation.

Initial labs showed thrombocytopenia, microcytic anemia, and hyponatremia. The total leukocyte count was 5.98 × 10³/mm³, with 30% neutrophils and 65% lymphocytes. Procalcitonin (PCT) was <0.05 ng/dL. CSF analysis revealed normal pressure, no red blood cells, a WBC count of 2 cells/μL (15% neutrophils, 58% lymphocytes), glucose of 58.8 mg/dL, and protein of 33 mg/dL (Table 1). Blood and CSF cultures were sent. The patient was started on levetiracetam and valproate (both at 27 mg/kg), empirical IV ceftriaxone (50 mg/kg), azithromycin, and vitamin B3.

**Table 1:** Laboratory results of the patient compared to reference values TLC: total leukocyte count, PCT: procalcitonin, RBC: red blood cells, WBC: white blood cells, CSF: cerebrospinal fluid, ↓: decreased level relative to reference range

Test	Result	Reference range
CBC		
TLC	5.98 × 10³/mm³	4.0-11.0 × 10³/mm³
Neutrophils	30%	40-60%
Lymphocytes	65%	20-40%
Platelet count	↓123× 10³/mm³ (thrombocytopenia)	150-450 × 10³/mm³
Hemoglobin	↓ 8 (microcytic anemia)	11.0-13.5 g/dL (for infants)
Serum electrolytes		
Sodium	↓ 120g/dL (hyponatremia)	135-145 mmol/L
Inflammatory marker		
PCT	<0.05 ng/mL	<0.5 ng/mL
CSF analysis		
Opening pressure	Normal	10-28 cm H₂O
RBC	0 cells/μL	0 cells/μL
WBC	2 cells/μL	0-5 cells/μL
Neutrophils in CSF	15%	0-6%
Lymphocytes in CSF	58%	40-80%
Glucose in CSF	58.8 mg/dL	40-70 mg/dL
Protein in CSF	33 mg/dL	15-45 mg/dL
Blood culture	Positive (day 2)	Not applicable
CSF culture	Negative	Not applicable

Audiological evaluation revealed mild right-sided sensorineural hearing loss (25-40 dB). Given the presence of transaminitis, hyponatremia, and thrombocytopenia, azithromycin (60 mg/kg daily) was added empirically to cover for possible scrub typhus. Serologies for scrub typhus IgM, toxoplasma IgM, CMV IgM, rubella IgM, dengue NS1, JE IgM, VDRL, and malaria were all negative.

**Figure 1:**
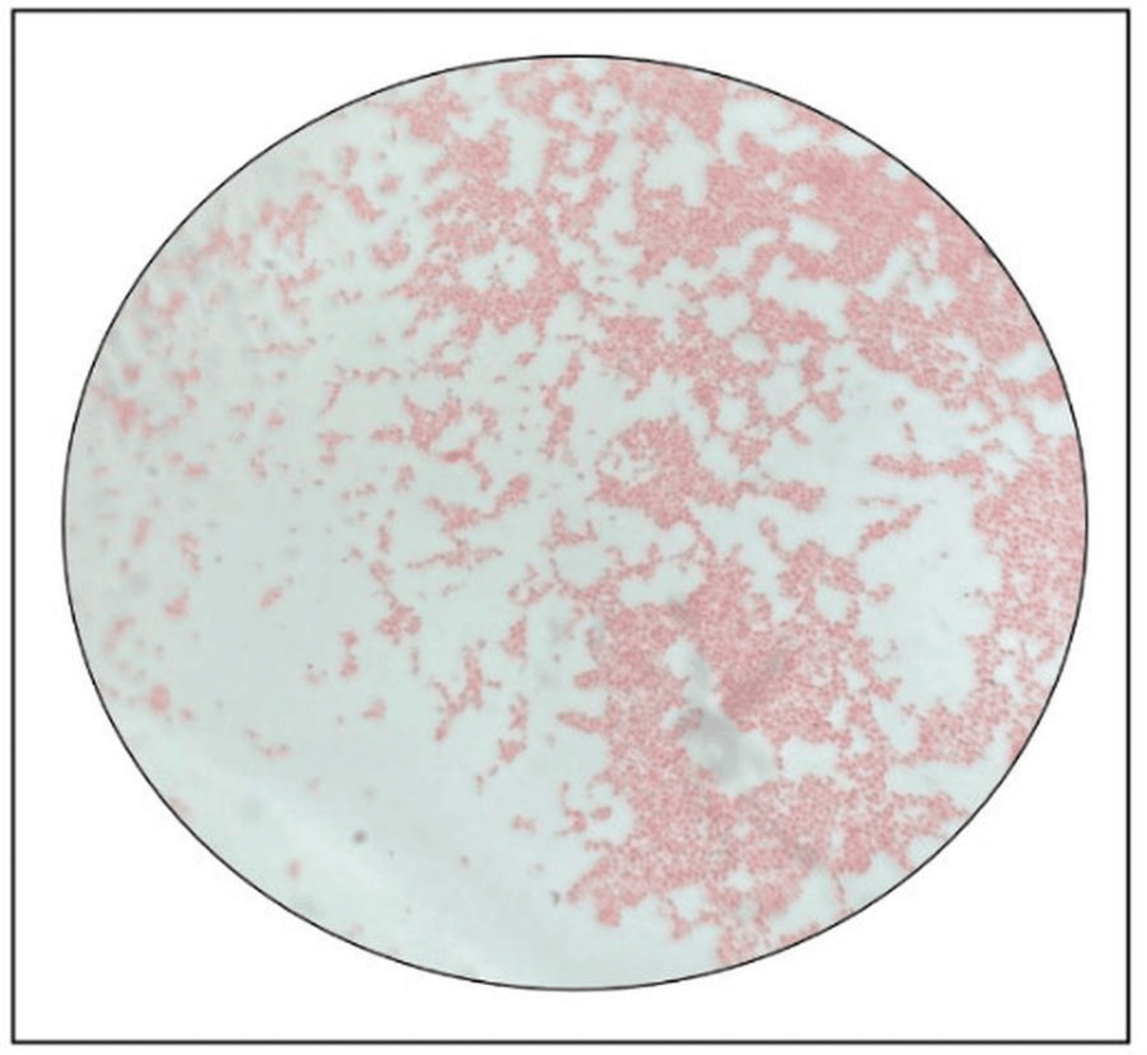
Gram stain of the isolate observed under 100× oil immersion objective The image shows numerous Gram-negative cocco bacilli appearing as short, pink-stained rods, arranged singly and in small clusters, consistent with the morphology observed in non-fermenting Gram-negative bacilli.

**Figure 2:**
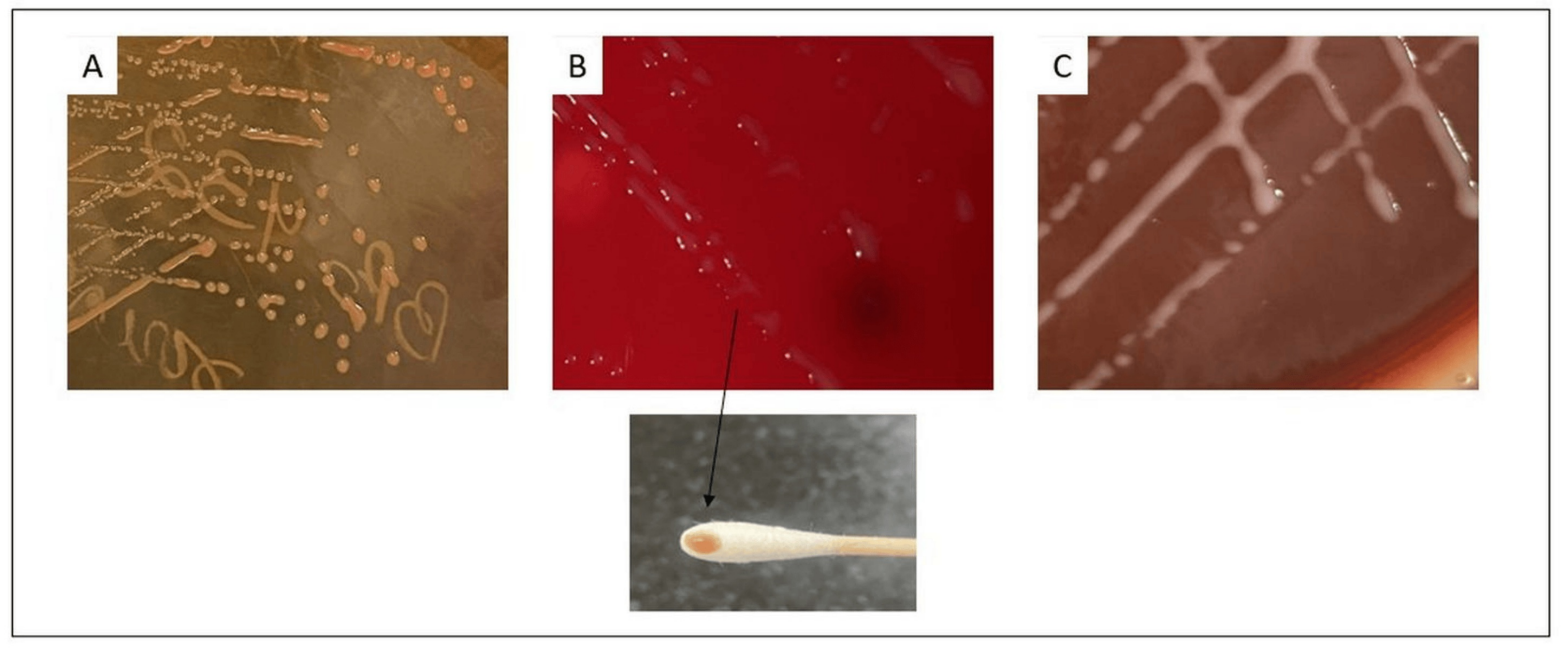
Colony morphology of the isolate on different culture media (A) Non-lactose fermenting, smooth, pink-pigmented colonies on MacConkey agar. (B) Growth on blood agar showing similar pink-pigmented colonies. The inset shows a colony picked using a sterile swab. (C) Growth on chocolate agar with distinct pink-pigmented colonies.

The child remained febrile, and the total leukocyte count rose to 15,060/mm³ by day 7. CSF culture was negative, but blood culture flagged positive after two days. Gram stain of the blood culture bottle showed Gram-negative coccobacilli (Figure 1), and a critical alert was issued. Colonies were 1-3 mm, round, translucent, and non-lactose fermenting on MacConkey agar (Figure 2A), and pink and non-hemolytic on blood and distinct pink-pigmented colonies on chocolate agar (Figure 2B-2C). The isolate was Gram-negative, motile, catalase- and oxidase-positive, citrate-utilizing, and urease-producing.

An initial report was sent indicating an unidentified Gram-negative bacillus, with antibiotic susceptibility extrapolated from *Pseudomonas aeruginosa*. The isolate was susceptible to ceftazidime, ceftriaxone, cefoperazone-sulbactam, cefepime, aztreonam, imipenem, meropenem, amikacin, gentamicin, and ciprofloxacin. A repeat blood culture on day three confirmed the same organism. Ceftriaxone was continued.

The organism was later identified as *R. gilardii* using the VITEK® 2 Compact system (BioMérieux, Marcy-l’Étoile, France) with 99% confidence. The patient showed clinical improvement, became afebrile, and had no further seizures. He was discharged after completing a seven-day course of ceftriaxone and azithromycin. The patient was advised to continue syrup Lavera (1 mL twice daily for one week), syrup Valparin, and vitamin D3, with follow-up in the pediatric outpatient department in one week and an audiology evaluation in two weeks.

## Discussion

*R. gilardii* infections are rare in humans, and they usually cause bacteremia, musculoskeletal infections, and skin and soft tissue infections. Bacteremia is the most common infection in the pediatric population (81.8%), and fever (100%) is the most common presentation [4]. Our patient also presented with fever along with seizure episodes.

*R. gilardii* infections are rare in humans, with bacteremia, musculoskeletal, and skin/soft tissue infections being the most common. Among pediatric cases, bacteremia accounts for 81.8%, with fever being the most frequent presentation (100%) [6]. Our patient similarly presented with fever and seizures.

*R. gilardii* can be isolated from soil, plants, freshwater, seawater, and hospital water systems, potentially leading to nosocomial infections such as catheter-associated bloodstream infections. Although its virulence factors are not well characterized, known mechanisms include biofilm formation, adhesion factors, extracellular enzymes (such as proteases and lipases), and β-lactamase-mediated antibiotic resistance [7].

Identification of *R. gilardii* typically requires methods beyond routine colony morphology and biochemical tests. Han et al. used 16S rRNA sequencing to characterize 36 *Roseomonas* isolates, finding that five were *R. gilardii*, while the others represented novel species [8]. In our case, identification was based on colony morphology, biochemical characteristics, and VITEK® 2 Compact analysis.

*Roseomonas* infections are not limited by age or sex. A review found that 92% of infected patients had central venous catheters (CVCs), and in 61% of cases, the organism was isolated from CVC-drawn blood samples [9]. In our case, the initial blood culture was positive for *R. gilardii*, and the same organism was later isolated from both CVC blood and catheter tip cultures. CVC removal, in addition to antibiotic therapy, likely contributed to the resolution of the infection.

Antibiotic resistance in *Roseomonas* species includes >90% resistance to penicillin and piperacillin/tazobactam, 77.8% resistance to cephalosporins, and <10% resistance to quinolones and carbapenems, with no reported resistance to aminoglycosides [6]. Despite the high cephalosporin resistance, our patient was treated with ceftriaxone due to clinical improvement, and therapy was continued.

## Conclusions

Bacteremia remains a significant cause of febrile illness in infants, and *R. gilardii* should be considered a potential, though rare, etiological agent. While mortality associated with *Roseomonas* infections is uncommon, prompt recognition and appropriate antimicrobial therapy are crucial for favorable outcomes. This case highlights the importance of heightened clinical awareness, accurate microbiological identification, and the systematic reporting of rare pathogens. Further studies and case reports are essential to expand our understanding of the clinical presentation, management, and outcomes of *R. gilardii* infections.
